# Whole-body cardiovascular MRI for the comparison of atherosclerotic burden and cardiac remodelling in healthy South Asian and European adults

**DOI:** 10.1259/bjr.20160342

**Published:** 2016-09

**Authors:** Jonathan R Weir-McCall, Deirdre B Cassidy, Jill J F Belch, Stephen J Gandy, J G Houston, Matthew A Lambert, Roberta C Littleford, Janice Rowland, Allan D Struthers, Faisel Khan

**Affiliations:** ^1^ Division of Cardiovascular and Diabetes Medicine, Ninewells Hospital, Dundee, UK; ^2^ NHS Tayside Medical Physics, Ninewells Hospital, Dundee, UK

## Abstract

**Objective:**

To determine the feasibility of using whole-body cardiovascular MRI (WB-CVMR) to compare South Asians (SAs)—a population known to have a higher risk of cardiovascular disease (CVD) but paradoxically lower prevalence of peripheral arterial disease—and Western Europeans (WEs).

**Methods:**

19 SAs and 38 age-, gender- and body mass index-matched WEs were recruited. All were aged 40 years and over, free from CVD and with a 10-year risk of CVD <20% as assessed by the adult treatment panel (ATP) III risk score. WB-CVMR was performed, comprising a whole-body angiogram (WBA) and cardiac MR (CMR), on a 3-T MRI scanner (Magnetom^®^ Trio; Siemens, Erlangen, Germany) following dual-phase injection of gadolinium-based contrast agent. A standardized atheroma score (SAS) was calculated from the WBA while indexed left ventricular mass and volumes were calculated from the CMR.

**Results:**

SAs exhibited a significantly lower iliofemoral atheroma burden (regional SAS 0.0 ± 0.0 *vs* 1.9 ± 6.9, *p* = 0.048) and a trend towards lower overall atheroma burden (whole-body SAS 0.7 ± 0.8 *vs* 1.8 ± 2.3, *p* = 0.1). They had significantly lower indexed left ventricular mass (46.9 ± 11.8 *vs* 56.9 ± 13.4 ml m^−2^, *p* = 0.008), end diastolic volume (63.9 ± 10.4 *vs* 75.2 ± 11.4 ml m^−2^, *p*=0.001), end systolic volume (20.5 ± 6.1 *vs* 24.6 ± 6.8 ml m^−2^, *p* = 0.03) and stroke volume (43.4 ± 6.6 *vs* 50.6 ± 7.9 ml m^−2^, *p* = 0.001), but with no significant difference in ejection fraction, mass-volume ratio or global functioning index. These differences persisted after accounting for CVD risk factors.

**Conclusion:**

WB-CVMR can quantify cardiac and atheroma burden and can detect differences in these metrics between ethnic groups that, if validated, may suggest that the paradoxical high risk of CVD compared with PVD risk may be due to an adverse cardiac haemodynamic status incurred by the smaller heart rather than atherosclerosis.

**Advances in knowledge:**

WB-CVMR can be used to stratify and compare disease between ethnicities.

## INTRODUCTION

Populations of South Asian (SA) origin and descent account for 78% of all deaths and 86.3% of all loss of disability-adjusted life years attributable to cardiovascular disease (CVD), with elevated risk observed both in the population in their native countries as well as in the global diaspora.^1,2^ Within Scotland, the SA population has a 16–53% higher risk of both coronary artery disease, cardiac failure and stroke incidence and mortality than the rest of the population.^3–5^ Despite this elevated risk for both coronary artery disease and cerebrovascular disease, a paradoxical lower incidence of peripheral arterial disease (PAD) has been observed.^6^ Given that coronary artery disease, stroke and PAD are all manifestations of atherosclerosis, the cause for this disparity is uncertain.

Previous studies have examined carotid plaque burden and coronary artery calcification differences between SAs and Europeans; however, a major limitation of these studies is that each of these examinations only gives information of a single vascular territory thus missing contemporaneous disease elsewhere within the body.^7–10^ Whole-body cardiovascular MRI (WB-CVMR) is a technique which comprises cardiac MR (CMR) and whole-body MR angiography (WB-MRA) sequences, allowing for the systemic assessment of whole-body stenotic atheroma burden, with cardiac structure, function and the detection of myocardial scarring, in a single examination. CMR combined with late gadolinium enhancement (LGE) is the gold standard for left ventricular quantification and detection of myocardial scarring and provides important clinical and prognostic information both in disease and in the general population.^11,12^ Pericardial adipose tissue, which is independently associated with the presence of coronary arterial disease, can also be quantified from routine CMR sequences.^13^ The atheroma score from WB-MRA has been shown to correlate well with traditional cardiac risk factors, the prevalence of obstructive coronary artery disease and future major adverse cardiovascular events.^14–17^ Thus, WB-MRA holds the potential for systemic evaluation of stenotic atheroma burden within an individual.

To date, neither CMR for the measurement of left ventricular mass, function and epicardial fat nor WB-MRA for the measurement of atheroma burden have been used to examine differences in cardiac function and atheroma burden between ethnic groups. We thus set out to determine the feasibility of using WB-CVMR to detect and compare early changes in left ventricular metrics and the atheroma burden in two separate ethnic groups.

## METHODS AND MATERIALS

### Ethics

The East of Scotland Research Ethics Committee approved the protocols (07/S1402/42). The study was conducted at Ninewells Hospital and Medical School, Dundee, UK, in accordance with the Good Clinical Practice Declaration of Helsinki. The volunteers gave written informed consent to participate in this study.

### Participants

Recruitment was from within the local community, running from June 2008 until February 2011 *via* general practitioner invitation or community-based recruitment. Recruitment criteria for the main study were as follows: (i) aged more than 40 years and (ii) free from known CVD or other indication for statin therapy as recommended by the Scottish Intercollegiate Guidelines Network report 97 (www.sign.ac.uk) published in February 2007. Exclusion criteria included: (i) pregnancy; (ii) known primary muscle disease; (iii) known atherosclerotic disease, including unstable angina, previous myocardial infarction, PAD, amputation, revascularization, hypertension, heart failure or cerebrovascular event; (iv) known diabetes; (v) active liver disease; (vi) other known illness or contraindication to MRI; (vii) participation in a clinical trial; (viii) inability to give informed consent; (ix) known alcohol abuse; and (x) blood pressure of >145/95 mmHg. SAs were defined as participants of Indian, Pakistani, Bangladeshi or Sri Lankan origin.

A total of 19 SAs were recruited, with 38 Western Europeans (WEs) randomly selected from the 1515 volunteers from the TAyside SCreening FOR Cardiovascular Events study, with these selected to be age, sex, height, weight and body surface area (BSA) matched to the SA cohort. Demographic data, anthropomorphic measures and blood samples were collected at the screening visit while the MRI scan was performed at the participants second study visit.

### MRI

Images were acquired as previously described.^18^ In brief, the scans were performed on a 32 radio frequency (RF) receiver channel, 3-T MRI scanner (Magnetom^®^ Trio; Siemens, Erlangen, Germany). For whole-body coverage, a combination of six RF coils, including head matrix, neck matrix, spine matrix, 2 body matrices and peripheral angiography phased-array RF surface coils, were used. The imaging protocol was carried out in four phases: (i) CMR cine sequences of the left ventricle (LV), (ii) MRA of the thoracic and neck, and distal lower limbs, (iii) LGE obtained after a delay of 10 min and (iv) MRA of the abdomen, pelvis and proximal lower limb.

#### Whole-body MR angiography protocol

WB-MRA images involved the acquisition of four overlapping three-dimensional (3D) data sets using a coronal spoiled FLASH sequence [repetition time (TR) = 2.6–3.47 ms; echo time (TE) = 0.96–1.21 ms; flip angle (FA) = 16–37°; pixel area = 1.1–1.5 mm^2^ and slice thickness = 1–1.4 mm, slight variation according to station and participant body habitus]. A dual injection, two-stage acquisition with uptitring of gadoteric acid contrast agent (Dotarem^®^; Guerbet, Villepinte, France) contrast volume was performed due to the benefits of this technique on image quality over a single injection technique.^19^ Stations 1 and 4 were acquired after an injection of 10 ml of gadoterate meglumine with a 20-ml saline flush. Following a delay of 15 min, during which the LGE sequences were obtained, Stations 2 and 3 were acquired following an injection of 15 ml of gadoterate meglumine with a 20-ml saline flush. All injections of contrast and saline were administered at 1.5 ml s^−1^.

#### Cardiac MR protocols

Left ventricular assessment involved the acquisition of a horizontal long axis, vertical long axis and stacked short axis cine images with repeated end-expiratory breath-holds, from the atrioventricular ring to the apex. These were acquired using a cine TrueFISP sequence with retrospective electrocardiographic gating (TR = 3.4 ms; TE = 1.48 ms; FA = 50–60°; pixel area = 1.4 × 1.9 mm^2^; slice thickness = 6 mm; interslice gap = 4 mm). 10 min after the injection of the first dose of contrast agent, the LGE images were acquired using a two-dimensional phase-sensitive inversion recovery sequence (TR = 846.4/5.21 ms; TE = 1.99 ms; FA = 20°; pixel area = 1.4 × 1.9 mm^2^; slice thickness = 6 mm; inter-slice gap = 4 mm, phases = 25).

#### Whole-body MR angiography image analysis

The 3D WB-MRA data sets were viewed offline (Carestream PACS Client Suite v. 10.1 sp1; Carestream Health, Rochester, NY) as source images using both multiplanar reconstruction and maximum intensity projections by one of four individuals experienced in WB-MRA reporting. Researchers were blind to the participants' ethnicity and clinical details during image analysis. The arterial network was divided into 31 vessel segments extending from the distal internal carotid arteries to the distal points of the calf vessels.^20^ Segments were scored according to the degree of narrowing of the lumen area, with stenosis graded at the narrowest part of the vessel. When more than one stenosis was present, the more severe (greater narrowing) of the two was used to score the vessel. Categorical scores from 0 to 4 were allocated to each vessel segment, where 0 = healthy segment with no stenosis, 1 = <50% stenosis, 2 = 50–70% stenosis, 3 = 71–99% stenosis and 4 = vessel occlusion. The “standardized atheroma score” (SAS) was calculated by summing each individual segment's stenosis score and divided by the number of diagnostic segments (*n*) before dividing by 4 which is the maximum potential score [Equation (1)] as described previously:^21^

(1) $$\text{SAS}\;=\;\;\left[\left(\frac{\sum \text{MRA }\;\text{score}}{n}\;\;\right)/4\;\right]\;\;\times \;\;100$$

The 31 vessel segments were subdivided into 5 anatomical territories: (i) the head and neck arteries, (ii) the aorta, (iii) the abdominal arteries, (iv) the iliofemoral arteries and (v) the run-off arteries in the lower limbs. Regional standardized atheroma scores were calculated for each anatomical territory.^22^

#### Cardiac MR image analysis

**Figure 1.**
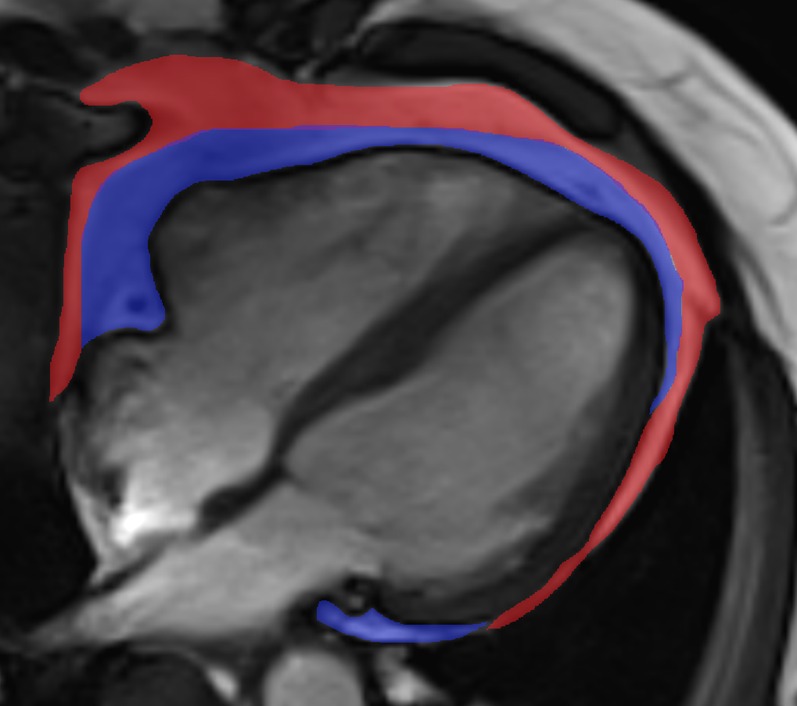
Four-chamber balanced steady state free precession image of the heart demonstrating the measurement of epicardial adipose tissue (blue) and paracardial adipose tissue (red) areas. SA, South Asian; WE, Western European. For colour image see online.

Left ventricular analysis images were analysed offline using commercial software (Argus, Siemens Multi-modality Work Platform v. VB15; Siemens) by 1 of 5 experienced researchers. Segmentation involved tracing endocardial and epicardial contours on the short-axis left ventricle images at end-diastolic and end-systolic phases of the cardiac cycle. The papillary muscles were treated as part of the blood pool volume unless they were indistinguishable from the myocardial wall, and then they were assigned as left ventricle muscle. Results were normalized to BSA using the DuBois formula.^23^ Left ventricular mass volume ratio (LVMVR) was calculated as Left ventricular mass (LVM)/left ventricular end diastolic volume (LVEDV). Left ventricular global function index (LVGFI) was calculated as LVGFI = [left ventricular stroke volume (LVSV)/LV global volume] × 100.^24^ Late gadolinium enhanced images of the left ventricle were inspected for evidence of myocardial signal enhancement using Carestream PACS Client Suite v. 10.1 sp1 (Carestream Health, Rochester, NY). Epicardial adipose tissue (EAT) and paracardial adipose tissue (PAT) were measured in the four-chamber balanced steady state free precession sequence at end diastole using Segment v. 1.9 R1917; (http://segment.heiburg.se).^25^ EAT was defined to be fat outwith the left ventricular myocardium and deep to the visceral pericardium. PAT was defined as the fat within the mediastinum but outwith the parietal pericardium (Figure 1). Thoracic adipose tissue was calculated by summing the EAT and PAT areas. Results were normalized to BSA using the Dubois formula. Reproducibility for both WB-MRA and CMR analyses have been previously described in detail.^18^

### Statistical methods

Data are expressed as mean ± standard deviation for continuous variables, median (interquartile range) for ordinal variables and *n* (%) for nominal variables. The SAS was heavily positively skewed and thus could not be transformed to a normal distribution, therefore only non-parametric tests were used for analysis of this variable. To test the null hypothesis that samples originate from the same distribution, an independent sample *t*-test was used for the parametric data, and a Kruskal–Wallis test by ranks was used for the non-parametric data. For categorical data, *χ*^2^ or Fisher exact were used as appropriate. Analysis of covariance was performed to confirm differences in cardiac volumes between the groups with the normalized LVM, end diastolic volume (EDV), end systolic volume (ESV) and LVSV each entered separately as the dependant variable correcting for age, sex, body mass index (BMI), blood pressure and smoking history. All data were analysed using SPSS^®^ statistical package v. 21.0 (IBM Corp., New York, NY; formerly SPSS Inc., Chicago, IL) and significance was assumed when *p* < 0.05.

## RESULTS

19 SAs (58% males, 51.4 ± 5.7 years) and 38 WEs (58% males, 50.8 ± 5.6 years) completed the study protocol. The demographic and CVD risk measures are documented in Table 1. Despite having similar BMI and BSA, SAs had a larger waist size than WEs (94 ± 11 *vs* 88 ± 10 cm, *p* = 0.04).

**Table 1.** The population demographics of the participants

Parameter	South Asian	Western European	*p*-value
*n*	19	38	
Male (%)	11 (58%)	22 (58%)	1
Age (years)	51.4 ± 5.7	50.8 ± 5.6	0.7
Heart rate	64.2 ± 8.0	60.1 ± 7.6	0.1
Systolic BP	120 ± 14	123 ± 11	0.5
Diastolic BP	73 ± 10	73 ± 10	0.9
Total cholesterol	5.1 ± 1.1	5.4 ± 1.2	0.3
LDL cholesterol (mmol l^−1^)	3.1 ± 0.8	3.4 ± 0.9	0.3
HDL cholesterol (mmol l^−1^)	1.1 ± 0.4	1.3 ± 0.3	0.08
Triglycerides (mmol l^−1^)	1.8 ± 0.9	1.8 ± 1.0	0.9
Fasting glucose	5.2 ± 1.0	5.1 ± 1.0	0.6
ATP III risk score	3.8 ± 3.5	3.6 ± 3.2	0.9
BMI (kg m^−2^)	27.8 ± 3.0	27.3 ± 2.9	0.5
BSA (m^2^)	1.9 ± 0.2	1.9 ± 0.2	0.2
Waist (cm)	94 ± 11	88 ± 10	**0.04**
Current/exsmoker (%)	3 (16%)	6 (16%)	1
SIMD	8 (7–8)	8 (5–9)	0.3
Family history of CVD	8 (26%)	16 (26%)	1

ATP, adult treatment panel; BMI, body mass index; BP, blood pressure; BSA, body surface area; CVD, cardiovascular disease; HDL, high-density lipoprotein; LDL, low-density lipoprotein; SIMD, Scottish Index of Multiple Deprivation.

Values expressed as mean ± standard deviation, median (inter quartile range) or *n* (%).

Bold values indicate *p* < 0.05.

The SA group had lower LVM, EDV, ESV and stroke volume (SV) (all *p* < 0.03; for full details, see Table 2). Despite these changes, there was no significant difference between the two groups in LV functional parameters with similar ejection fraction, LVMVR and LVGFI (all *p* > 0.6; for full details, see Table 2). Neither group exhibited any evidence of myocardial scarring on LGE imaging. On analysis of covariance, the differences between the groups for LVM, EDV, ESV and SV all persisted after accounting for age, sex, BMI, systolic and diastolic blood pressure and smoking status (analysis of covariance results: LVM *p* = 0.001; EDV *p* < 0.001; ESV *p* = 0.028; SV *p* = 0.001). WEs had a significantly higher volume of PAT (7.4 ± 6.2 *vs* 5.0 ± 2.0 cm^2^ m^−2^, *p* = 0.04). There was no difference in EAT (2.7 ± 1.7 *vs* 2.6 ± 1.4 cm^2^ m^−2^, *p* = 0.8) or thoracic adipose tissue volume (10.1 ± 7.6 *vs* 7.6 ± 2.8 cm^2^ m^−2^, *p* = 0.09) between the two groups.

**Table 2.** Comparison of cardiac MRI metrics between the two groups

Parameter	South Asian	Western European	*p*-value
LVM (g m^−2^)	46.9 ± 11.8	56.9 ± 13.4	**0.008**
LVEDV (ml m^−2^)	63.9 ± 10.4	75.2 ± 11.4	**0.001**
LVESV (ml m^−2^)	20.5 ± 6.1	24.6 ± 6.8	**0.028**
LVSV (ml m^−2^)	43.4 ± 6.6	50.6 ± 7.9	**0.001**
LVEF (%)	68.3 ± 5.9	67.5 ± 6.6	0.68
CI (l min^−1^ m^2^)	5.06 ± 7.9	6.14 ± 9.8	0.6
LVMVR (g ml^−1^)	0.79 ± 0.14	0.78 ± 0.16	0.9
LVGFI	49.2 ± 7.9	48.5 ± 6.5	0.7
LGE (%)	0 (0)	0 (0)	1
EAT (cm^2^ m^−2^)	2.6 ± 1.4	2.7 ± 1.7	0.8
PAT (cm^2^ m^−2^)	5.0 ± 2.0	7.4 ± 6.2	**0.04**
TAT (cm^2^ m^−2^)	7.6 ± 2.8	10.1 ± 7.6	0.09
EAT/TAT	0.34 ± 0.1	0.29 ± 0.09	0.12

CI, cardiac index; EAT, epicardial adipose tissue; LVEDV, indexed left ventricular end diastolic volume; LVEF, left ventricular ejection fraction; LVESV, indexed left ventricular end systolic volume; LVM, left ventricular mass; LVMVR, left ventricular mass volume ratio; LVGFI, left ventricular global function index; LVSV, indexed left ventricular stroke volume; LGE, late gadolinium enhancement; PAT, paracardial adipose tissue; TAT, thoracic adipose tissue.

Values expressed as mean ± standard deviation or *n* (%).

Bold values indicate *p* < 0.05.

**Figure 2.**
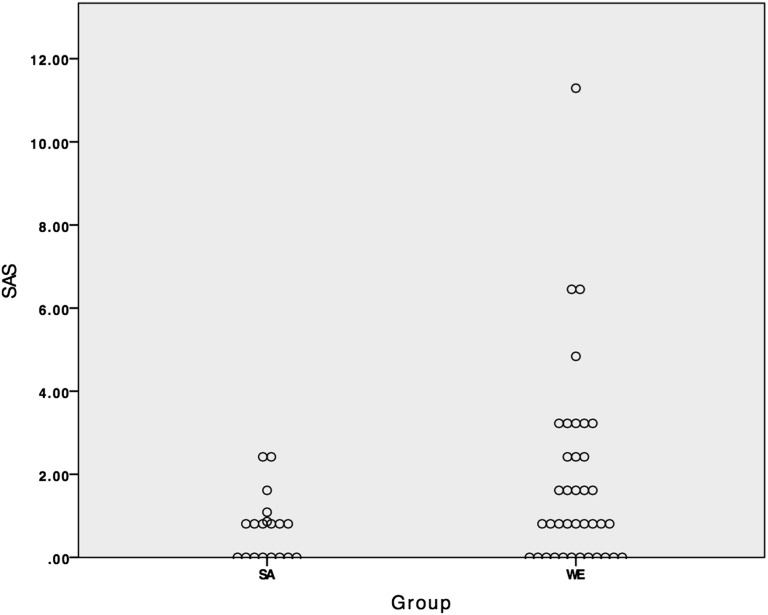
Scatter plot comparing whole-body standardized atheroma score (SAS) between the South Asian (SA) and Western European (WE) cohorts.

On whole-body angiography, the majority of the vessels had no evidence of disease, with 97% of vessels in the SA group and 95% of the vessels in WE group demonstrating no evidence of stenotic atheroma formation (*p* > 0.05). The iliofemoral segments showed a significant difference between the two groups with a higher local SAS in this region in the WE cohort (1.9 ± 6.9 *vs* 0.0 ± 0.0, *p* = 0.048). Additionally, there was a trend towards a lower global atheroma burden in the SA population than the WE cohort (whole-body SAS 0.7 ± 0.8 *vs* 1.8 ± 2.3, *p* = 0.10) (Figure 2). This effect was not site specific with a similar pattern seen in all five of the anatomical vascular territories (Table 3). These differences were caused by a higher incidence of mild (1–49%) and moderate stenosis (50–70%) in the WEs although neither of these reached significance (*p* = 0.10 and *p* = 0.08, respectively).

**Table 3.** Comparison of MR angiography metrics between the two groups

Parameter	South Asian	Western European	*p*-value
WB SAS	0.7 ± 0.8	1.8 ± 2.3	0.10
Head/neck SAS	0.5 ± 1.1	1.9 ± 4.0	0.21
Aorta SAS	0.9 ± 2.6	1.3 ± 3.1	0.59
Abdomen SAS	1.8 ± 3.8	3.2 ± 4.4	0.26
Iliofemoral SAS	0.0 ± 0.0	1.9 ± 6.9	**0.048**
Run-off SAS	0.7 ± 2.2	0.9 ± 2.9	0.77
Normal vessels	501 (97%)	1117 (95%)	0.2
Vessels with 1–50% stenosis	14 (3%)	46 (4%)	0.1
Vessels with 51–70% stenosis	1 (0.2%)	9 (0.8%)	0.08
Vessels with 71–99% stenosis	0	3 (0.2%)	0.3
Occluded vessels	0	1 (0.01%)	0.3

SAS, standardized atheroma score; WB, whole-body.

Values expressed as mean ± standard deviation, median (inter quartile range) or *n* (%).

Bold value indicates *p* < 0.05.

## DISCUSSION

In this study, we have shown that WB-CVMR is a useful and feasible technique for the comparison of cardiac function and atheroma burden between different ethnic groups. Although this was a preliminary study and the group sizes were small, this technique was able to demonstrate for the first time that healthy SAs have a lower incidence of atherosclerosis in the iliofemoral vessels and smaller hearts compared with WEs. These findings demonstrate that even in those individuals with a low prevalence of risk factors, early disease can be witnessed in a pattern that might start to explain the paradoxically low incidence of PAD in SAs compared with their markedly elevated risk of ischaemic heart disease.

The finding of lower LV volumes and mass in SAs is consistent with previous echocardiographic studies using both two-dimensional and 3D echo, providing supportive evidence for the validity of the findings of the technique.^26,27^ Despite significant differences in the left ventricular volumes and mass, there were no differences in ejection fraction, mass : volume ratio or left ventricular global function index. Both LVMVR and LVGFI are measures of LV function that encompass both cavity dimension and myocardial remodelling and are better prognostic markers of incident cardiac events and stroke than LV mass or volumes.^11,24^ Given that these values are preserved in the SA cohort indicates that despite much smaller ventricular dimensions, there is continued efficient functionality of the left ventricle. LV mass is known to correlate strongly with lean body mass which is known to be lower in SAs than WEs, thus some of the observed differences may be due to differences in body composition.^27,28^ The observation that the SA cohort had a larger waist size in our current study despite equivalent mass and BMI would certainly suggest a higher percentage of fat mass and thus lower lean body mass within the cohort which could explain the discrepancy in mass between the two groups. Despite lower lean body masses and, therefore in theory, lower LV mass as a result of this, a previous study by Chahal et al^29^ demonstrated the incidence of LVH and concentric LV remodelling, both compensatory responses to LV wall stress, to be increased in SAs compared with Europeans. Thus it may be hypothesized that the smaller hearts which are having to produce the same cardiac output as the larger WE hearts are put under adverse ventricular wall stress. Indeed, diastolic function and ventricular relaxation rates, markers of left ventricular fibrosis and stiffening are also impaired in SAs compared with WEs.^27^ However, further work in this area is warranted to further evaluate and extricate the comparative effects of lean body mass, wall stress and fibrosis.

However, evidence supporting either of these potential causative mechanisms is limited but is an interesting avenue for future work.

In our current study, we have demonstrated for the first time a significantly greater burden of stenotic arterial disease within the pelvic vessels of WEs compared with SAs and a trend towards greater global atheroma burden. These findings will require validation in a larger cohort and provide the preliminary work required to justify such a study. Despite the small group numbers involved, there are several reasons that increase the likelihood of these results representing the true differential burden of disease between populations. Prior studies, which have used ankle brachial pressure index as a surrogate marker for the presence of peripheral vascular disease, have demonstrated a reduced prevalence of PAD in SAs, and a poor concordance of ankle brachial pressure index with the presence of coronary artery disease status within the SA populace, both of which would support the observation of a lower atherosclerotic burden within the lower limb vasculature.^30,31^ Although previous studies have demonstrated similar carotid intima media thickness (CIMT) and plaque volume in SAs and Europeans,^7,8^ the discordance between similar CIMT in SAs and Europeans and our current findings of higher peripheral vascular disease in WEs can at least be partly explained by the relatively poor concordance of CIMT with global atheroma burden.^32,33^

Despite similar height, weight, BMI and BSA, the SA group had a larger waist size which is a known risk factor for future diabetes and cardiovascular events.^34^ A previous study has demonstrated a positive correlation between visceral adiposity and atheroma score using whole-body angiography, and thus the findings observed in the SA group are contrary to expectations.^35^ In addition, despite this elevation in waist size, commensurate increase in the EAT volume was not observed, in fact the opposite was observed within the paracardial fat, with the WE population exhibiting a greater volume of paracardial fat. Epicardial fat has been described to be a risk factor for coronary artery disease due to its perivascular release of chemokines and cytokines,^13^ thus the lack of differences between the populations removes this as a potential source of the regional variation in plaque formation. Paracardial fat has also been described as a risk factor for the development of diabetes and CVD,^36^ thus the observed higher volumes within the WE population than the SA population is contrary to expectations and again points away from this as a possible contributory factor to explain the higher incidence of diabetes and cardiovascular events in the SA populace.

The strengths of the current study are its novelty and its simultaneous assessment of the cardiac and vascular network in a single examination. There are, however, several limitations. The sample size is small and the study is single centred, limiting the generalizability of the results, thus replication in a larger multicentre cohort is required with the current study providing an important first step in the justification for such work. WB-MRA is a lumenographic technique, thus extraluminal plaque formation will be missed, potentially underestimating atheroma burden in the populations. However, whilst techniques such as black blood imaging may hold the potential for analysis of early wall thickening and stenosis assessment, this technique has yet to be used and validated in whole-body vascular assessment, unlike the current angiographic technique. The voxel size of the lower limb angiography station is such that assessment of the more distal smaller calibre arteries was challenging and could thus underestimate infrageniculate disease as is seen in the diabetic population.^37^ Information on exercise was not available within the current study thus confounding effects of differential exercise patterns (which are known to affect LV metrics) between the two cohorts cannot be excluded. Finally, the differences between the cardiac mass and volumes are calculated after indexing to the BSA. Observed differences could therefore be secondary to inappropriate allometric normalization from BSA calculations which were derived in a white population, especially given the larger waist size in the SA cohort. However, given that the height, weight and BMI are matched between the cohorts and that similar results were observed using the raw LV data, the effect of this is likely to be negligible, especially given the magnitude of the differences between the groups.

## CONCLUSION

WB-CVMR can quantify cardiac and atheroma burden and can detect differences in these metrics between ethnic groups that, if validated, suggests that the paradoxical high risk of CVD compared with PVD risk may be due to an adverse cardiac haemodynamic status incurred by the smaller heart rather than atherosclerosis.

## FUNDING

The present study was funded by the Souter Charitable Foundation and the Chest, Heart and Stroke Scotland Charity. JRWM is supported by the Wellcome Trust through the Scottish Translational Medicine and Therapeutics Initiative (grant no. WT 085664) in the form of a clinical research fellowship. Neither group had any role in study design, the collection, analysis and interpretation of data; in the writing of the article; nor in the decision to submit the article for publication.
